# Oxytocin Motivates Non-Cooperation in Intergroup Conflict to Protect Vulnerable In-Group Members

**DOI:** 10.1371/journal.pone.0046751

**Published:** 2012-11-07

**Authors:** Carsten K. W. De Dreu, Shaul Shalvi, Lindred L. Greer, Gerben A. Van Kleef, Michel J. J. Handgraaf

**Affiliations:** 1 Department of Psychology, University of Amsterdam, Amsterdam, The Netherlands; 2 Department of Economics, Wageningen University, Wageningen, The Netherlands; The University of South Wales, Australia

## Abstract

Intergroup conflict is often driven by an individual's motivation to protect oneself and fellow group members against the threat of out-group aggression, including the tendency to pre-empt out-group threat through a competitive approach. Here we link such defense-motivated competition to oxytocin, a hypothalamic neuropeptide involved in reproduction and social bonding. An intergroup conflict game was developed to disentangle whether oxytocin motivates competitive approach to protect (i) immediate self-interest, (ii) vulnerable in-group members, or (iii) both. Males self-administered oxytocin or placebo (double-blind placebo-controlled) and made decisions with financial consequences to themselves, their fellow in-group members, and a competing out-group. Game payoffs were manipulated between-subjects so that non-cooperation by the out-group had high vs. low impact on personal payoff (personal vulnerability), and high vs. low impact on payoff to fellow in-group members (in-group vulnerability). When personal vulnerability was high, non-cooperation was unaffected by treatment and in-group vulnerability. When personal vulnerability was low, however, in-group vulnerability motivated non-cooperation but only when males received oxytocin. Oxytocin fuels a defense-motivated competitive approach to protect vulnerable group members, even when personal fate is not at stake.

## Introduction

Intergroup relations are often marked by non-cooperation and competitive behavior, creating levels of aggression and violence that bring about severe societal costs [1]–[3]. Intergroup hostilities have been traced back to the greedy desire among members of one group to subordinate rivaling out-groups and acquire their resources [4], [5], and/or the defensive motivation to serve and protect the in-group against possible out-group threat [6]–[8].

Competitive approach of, and defense-motivated non-cooperation towards rivaling out-groups serves the individual's personal interests, as it signals the individual's loyalty and commitment to fellow group members, encouraging inclusion rather than exclusion from profitable within-group exchange and cooperation [5], [9]–[11]. At the same time, defense-motivated non-cooperation serves group-interests because it may pre-empt possible attack by rivaling out-groups and may deter them from aggressing against the in-group [7], [12]. Accordingly, group-living animals such as Siberian Jays and Meerkats aggress against rivals and predators especially in the presence of offspring, and such parental mobbing increases offspring survival and provides for group formation and cooperative kin-societies [13]–[15]. In humans, such parochial altruism has likewise been associated with group survival and prosperity [4], [11].

Because of its functionality to individual and group survival, the human brain may have evolved neurobiological mechanisms that support and sustain defense-motivated non-cooperation towards rivaling out-groups [16]–[18]. Here we examine this possibility by focusing on oxytocin, an evolutionary ancient neuropeptide that is produced in the hypothalamus and functions as hormone and neurotransmitter [19], [20]. In humans, oxytocin projects into the amygdala, hippocampus, and regions of the spinal cord that regulate the parasympathic branch of the autonomic nervous system [21], [22]. It attenuates stress responses [23]–[25], and reduces amygdala activation and its coupling to brainstem centers responsible for autonomic and behavioral components of fear [26]–[28]. Furthermore, oxytocin interacts with dopaminergic, reward processing areas [29]–[31], that may motivate cooperation towards reciprocating others [32]. Indeed, at the behavioral level, oxytocin reduces betrayal aversion [26], [33], and motivates trust and cooperation especially when others are familiar [34], not untrustworthy [35], and belonging to one's own group [17], [36].

Three lines of evidence suggest that oxytocin not only promotes cooperation, but is involved also in defense-motivated non-cooperation towards rivaling out-groups. In free-living meerkats, infusion of oxytocin versus placebo motivated an array of cooperative behaviors including longer time-on-guard [37], in lactating rats bred for high anxiety it motivated maternal aggression against virgin intruders [38], and in breast-feeding mothers plasma oxytocin predicted hostility towards a female stranger [39]. In humans, intranasal oxytocin versus placebo motivated more positive evaluations of the in-group compared to rivaling out-groups [40], and non-cooperation towards rivaling out-groups especially when out-group threat was high [17], [41]. However, because in these studies the individual's self-interest perfectly correlated with in-group interests, it is unknown whether, in intergroup competition, oxytocin motivates protection of (i) immediate self-interest, (ii) vulnerable in-group members, or (iii) some combination of both.

Disentangling these different possibilities would advance our understanding of the role of oxytocin in the evolution of group life. If, in intergroup conflict, oxytocin-motivated competitive approach varies as a function of the extent to which self-interest is at stake, we have to re-interpret previous work as showing that oxytocin up-regulates what Adam Smith called “enlightened self-interest.” But if oxytocin-motivated competitive approach varies (also) as a function of the extent to which the interests of fellow in-group members is at stake, we may be in a position to infer that oxytocin shifts the individual's focus away from immediate self-interest and towards in-group interests, and that oxytocin's functions include a tendency to tend-and-defend the in-group.

To disentangle these possibilities, we developed a behavioral game in which participants acted as the representative of a group of three that competed with a protagonist representing a three-person out-group. Cardinal payoffs in the game were manipulated so that non-cooperation by the out-group had strong versus weak impact on the participant's personal outcome (personal vulnerability) and, independently, the outcomes for the two other in-group members that were represented by the participant (in-group vulnerability). We expected to replicate earlier work [17], [36] showing that intranasal administration of oxytocin rather than placebo (i) motivates tending for the in-group. We tested the new hypotheses that even when personal vulnerability is low, oxytocin (ii) motivates in-group protection; and (iii) leads to non-cooperation towards the out-group protagonist, especially when in-group vulnerability is high.

### Methods Overview

In keeping with past work [26], [27], hypotheses were tested in a double-blind placebo controlled between-subjects design, with 102 males self-administering 24 IU oxytocin or placebo through nasal spray. After the standard loading time of forty minutes [26], participants were, on the basis of a trivial criterion, assigned to a three-person group and informed that they would engage in a decision making task with members of another three-person group for real money (Materials and Methods) [17]. Participants were paired to a member of the other group, told that they would simultaneously decide to cooperate (option A) or not (option B), and that decisions accrued financial earnings to themselves, the two other in-group members they represented, and to the out-group representative and his constituency.

**Figure 1:**
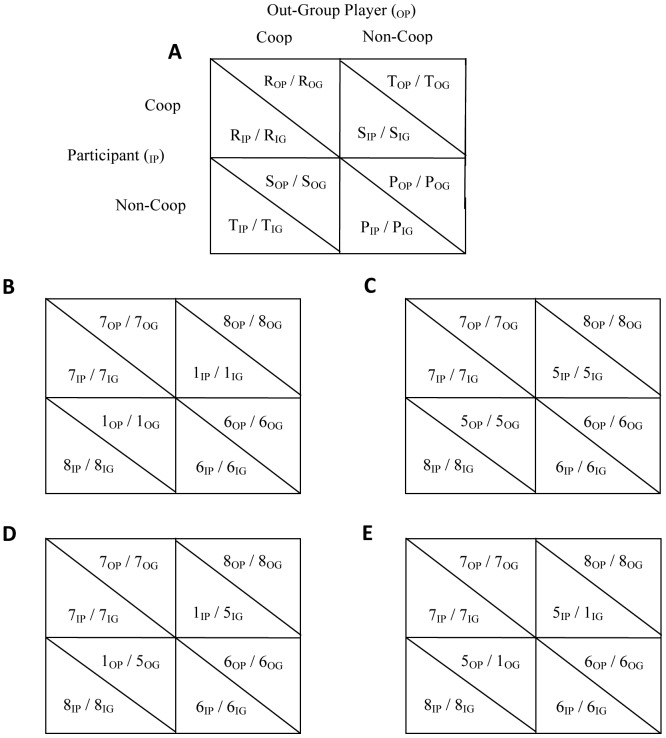
Game Structures used in the Experiment. (**A**). Between-Group Prisoner's Dilemma (BG-PD) with T(emptation)>R(eward)>P(unishment)>S(ucker); subscript IP (In-Group Player) are payoffs to the participant; subscript IG (In-Group) are payoffs to other in-group members; subscript OP (Out-group Player) are payoffs to the out-group protagonist; subscripts OG (Our-Group) are payoffs to other out-group members. Participant (row player) and Out-Group Player (column player) decide between coop (cooperation) and non-coop (non-cooperation); (**B**). Payoff Structure in the High Personal Vulnerability/High In-Group Vulnerability Condition; (**C**). Payoff Structure in the Low Personal Vulnerability/Low In-Group Vulnerability Condition; (**D**). Payoff Structure in the High Personal Vulnerability/Low In-Group Vulnerability Condition; (**E**). Payoff Structure in the Low Personal Vulnerability/Low In-Group Vulnerability Condition.

The game was modeled after the Prisoner's Dilemma (PD; Fig. 1A) so that participant's decision combined with the out-group representative's decision yields four possible payoffs to all individuals involved (i.e., participant, his in-group members, the out-group representative, and his out-group members): Temptation (T), Reward (R), Punishment (P), and Sucker (S), which are ordered as T>R>P>S [42], [43]. Fig. 1B shows that if both participant and out-group representative cooperate, both in-group and out-group obtain the Reward payoff of 7, which exceeds the Punishment payoff for mutual non-cooperation of 6 (i.e., [R−P] = [7−6] = 1 (henceforth Cooperator's Gain). The dilemma occurs because both participant and out-group representative obtain even higher payoffs for their own group by non-cooperation. Non-cooperation may reflect the greedy desire to exploit the out-group, and/or the defensive desire to protect the in-group against out-group non-cooperation. First, if the out-group were to cooperate, participants obtain higher outcomes for their in-group by non-cooperation (T) than by cooperation (R) (in Fig. 1B: [T−R] = [8−7] = 1; henceforth Greed). Second, if the out-group were to non-cooperate, participants obtain higher outcomes for their in-group by non-cooperation (P) than by cooperation (S) (in Fig. 1B: [P−S] = [6−1] = 5; henceforth Fear).

In Fig. 1B, Fear to participant as in-group representative (henceforth Personal Vulnerability) is at the same level as Fear to other in-group members (henceforth In-Group Vulnerability). By manipulating cardinal payoffs [17], [43], we varied Personal Vulnerability independently from In-Group Vulnerability (Fig. 1B—Fig. 1E). In Fig. 1B and 1D, Personal Vulnerability is high at [P−S] = [6−1] = 5, whereas it is low at [P−S] = [6−5] = 1 in Fig. 1C and 1E. In Fig. 1B and 1E, In-Group Vulnerability is high at [P−S] = [6−1] = 5, whereas In-Group Vulnerability is low at [P−S] = [6−5] = 1 in Fig. 1C and 1D. Because Cooperator's Gain and Greed are constant across games, higher non-cooperation in Fig. 1B and 1D compared to Fig. 1C and 1E must reflect a desire to protect oneself against a possibly non-cooperative out-group representative; higher non-cooperation in Fig. 1B and 1E compared to Fig. 1C and 1D must reflect a desire to protect one's in-group against a possibly non-cooperative out-group representative. Thus, self-protection as a motive for non-cooperation is stronger when the out-group representative's decision has strong rather than weak impact on personal outcomes (Fig. 1B and D versus Fig. 1C and E); in-group-protection as a motive for non-cooperation is stronger when the out-group representative's decision has strong rather than weak impact on outcomes to in-group members (Fig. 1B and E versus Fig. 1C and D).

Following instructions using the games in Fig. 1B—1E (depending on condition; Materials and Methods), participants made five anonymous choices without feedback about their protagonist's choice, were asked each time to accurately assess how often their protagonist made a non-cooperative choice (range 0–5), and answered questions about their reasons for non-cooperation (in-group tending, protectionism) (Materials and Methods).

## Results

**Figure 2:**
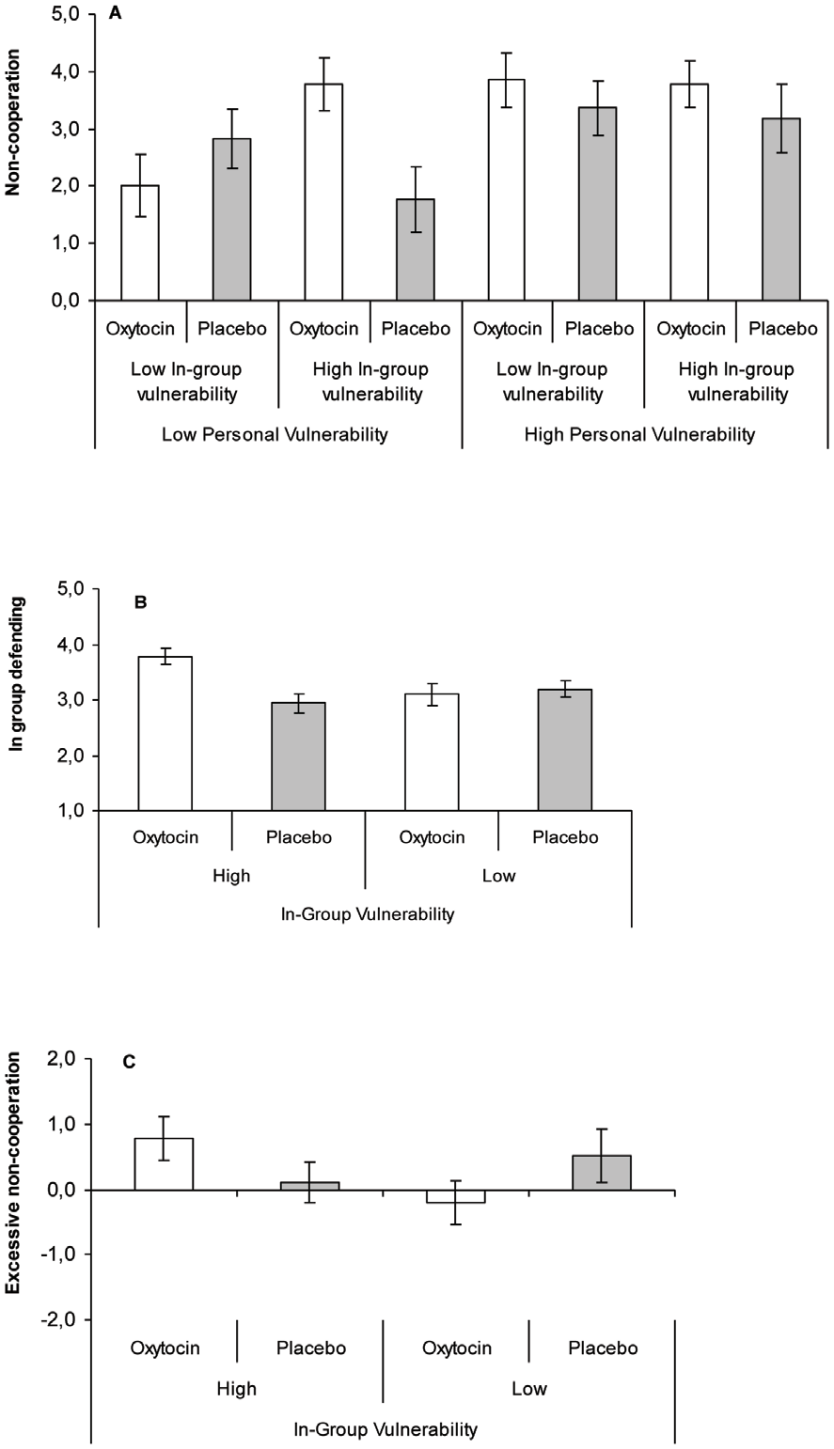
Non-Cooperation, In-Group Defending, and Excessive Non-Cooperation. (**A**). Under low personal vulnerability, oxytocin produces more non-cooperation than placebo when in-group vulnerability is high rather than low (range 0–5, displayed ±SE). (**B**). Compared to placebo, oxytocin enhances motivation to defend the in-group when in-group vulnerability is high rather than low (based on *N* = 72; range 1–7, displayed ±SE). (**C**). Excessive non-cooperation (i.e., own non-cooperation>expected out-group non-cooperation; range −5 to +5; displayed ±SE) emerges under high in-group vulnerability when individuals received oxytocin rather than placebo.

All data were analyzed in 2 (Treatment: Oxytocin/Placebo)×2 (Personal Vulnerability: High/Low)×2 (In-Group Vulnerability: High/Low) between-subjects ANOVAs. For non-cooperation there was a main effect for Personal Vulnerablity, *F*(1,94) = 7.86, *p* = 0.006, partial η^2^ = 0.076, a trend towards an interaction between In-Group Vulnerability and Treatment, *F*(1,94) = 3.19, *p* = 0.077, and a significant three-way interaction, *F*(1,94) = 5.97, *p* = 0.016, partial η^2^ = 0.06. Fig. 2A shows relatively high levels of non-cooperation when Personal Vulnerability was high, and non-cooperation was not influenced by Treatment, In-group Vulnerability, or their interaction, *F*s<1. When Personal Vulnerability was low, however, non-cooperation was higher when In-Group Vulnerability was high rather than low, but only among individuals given oxytocin rather than placebo (In-group Vulnerability×Treatment interaction, *F*[1,99] = 9.10, *p* = 0.003; Fig. 2A).

Oxytocin-induced non-cooperation appears motivated by the desire to protect vulnerable in-group members, as predicted in hypothesis (iii). Indeed, as predicted in hypothesis (i), in-group tending was higher when participants received oxytocin rather than placebo, *M* = 3.828 vs. *M* = 3.002, *F*(1,94) = 7.23, *p* = 0.008, partial η^2^ = 0.071 (all other *F*<3.00, p>0.10) In-group defending was measured in 72 participants (Methods & Materials), and was predicted by a significant Treatment×In-Group Vulnerability interaction, *F*(1,64) = 7.18, *p*<0.010, partial η^2^ = 0.093. Fig. 2B shows that, regardless the level of personal vulnerability, in-group defending is higher when in-group vulnerability is high rather than low, but only among individuals who received oxytocin, *F*(1,69) = 6.18, *p*<0.015 rather than placebo, *F*(1,69) = 1.89, *p*<0.178. Further supporting hypothesis (ii), in-group defending positively related to non-cooperation, *r*(72) = 0.428, *p*<0.001.

The game structure was symmetrical (see Fig. 1B–E), and participant non-cooperation may have been (also) motivated by condition-dependent expectations of the out-group representative's cooperativeness. However, ANOVA revealed no effects on expectations (all *F*[1,94]<1.41, *p*s>0.238, partial η^2^<0.015; *M*_overall_ = 2.873, *SD* = 1.978): Oxytocin's effects on participants' non-cooperation cannot be attributed to altered expectations of out-group behavior.

To explore whether in-group protection creates excessive non-cooperation, we analyzed the difference between participant non-cooperation and expected out-group non-cooperation. This revealed, first, more excessive non-cooperation when personal vulnerability was high (*M* = 0.715) rather than low (*M* = −0.095), *F*(1,94) = 5.59, *p* = 0.020, partial η^2^ = 0.056. Second, we observed a Treatment by In-Group Vulnerability interaction, *F*(1,94) = 4.14, *p*<0.045, partial η^2^ = 0.042 (all other *F*<1.51, all *p*>0.22) (see Fig. 2C). When given placebo, participants matched the level of expected out-group non-cooperation, whether in-group vulnerability was high (*M* = 0.115) or low (*M* = 0.522), *F*(1,99) = 0.65, *p* = 0.424. When given oxytocin, however, participants' level of non-cooperation was higher when in-group vulnerability was high (*M* = 0.778) rather than low (*M* = −0.192), *F*(1,99) = 4.01, *p* = 0.048. In fact, and as shown in Fig. 2C, only when in-group vulnerability was high and participants received oxytocin did the difference between own non-cooperation and expected out-group non-cooperation differ from 0 (one-sample *t*[27] = 2.294, *p*<0.030; all other *t*<|1.199|, *p*s>.243). Thus, males engaged in excessive non-cooperation when in-group vulnerability was high and they received oxytocin rather than placebo.

## Discussion

The neuropeptide oxytocin not only motivates cooperation towards familiar others, and those belonging to one's in-group [17], [34], [36], it also drives vigilance and time-on-guard [37] as well as non-cooperation towards rivaling out-groups [17], [41]. Through a newly developed inter-group game we clarified that earlier findings should not be interpreted as if oxytocin increases the individual's motivation to serve personal interests. Rather, findings here reveal that oxytocin-motivated non-cooperation is driven by the desire to protect vulnerable in-group members, even when immediate self-interest is not at stake. The male participants in our study behaved as the proverbial Mamma Bear who, while not being immediately in danger herself, lashes out against predators threatening her cubs. As predicted, such protective behavior emerged especially when participants received oxytocin rather than placebo.

Because groups were ad hoc and formed on the basis of a trivial criterion the (vulnerable) individuals protected here were relative strangers and not genetically related to the participant, findings are at odds with kin-selection theory of altruism that predicts altruism to be close to absent for genetically unrelated others [44]. Findings are consistent, however, with recent advances in natural selection theory on (human) eusociality [45], [46]. In essence, the idea is that altruism benefits group survival and that humans—like other eusocial species such as ants and honeybees—have evolved capacity to quickly learn who does, and does not, belong to the in-group and learned to act on those insights to further group (rather than personal) survival and prosperity. Extensive work in social psychological science further attests to the fact that humans are highly flexible in creating cognitive representations of in-group versus out-group, and act upon those social categorizations quickly by favoring the in-group relative to the out-group [47], [48]. As shown here, and elsewhere [17], [36], [40], [49], oxytocin appears pivotal in up-regulating the human response to (arbitrary) in-group/out-group distinctions, shifting the focus from protecting and promoting oneself towards protecting and promoting the (members of the) in-group [50].

There is no evidence that oxytocin motivates greed and spiteful tendencies towards rivaling out-groups [17], [36]. The behavioral decision game developed here thus varied fear parameters and held constant greed and cooperator's gain. The game structure can be altered, however, to create varying levels of greed too. One avenue for new research is to study neurohormonal influences on greed-driven non-cooperation towards rivaling out-groups. Whereas oxytocin may be unrelated to greed, we surmise that such exploitative non-cooperation may be a function of vasopressin and testosterone, neurohormones shown to be involved in aggression and dominance seeking [51], [52].

Whether self-interest and the interest of fellow in-group members can be truly separated may be debatable. For example, human and non-human cooperation may be explained as serving the individual's long-term personal interests in survival and (re)productive success, and defense-motivated non-cooperation towards an out-group accrues quality signals to the individual that provide long-term benefits [5], [9], [10], [11]. Rather than attempting to solve this deep philosophical question, we approached this issue by creating varying degrees to which the out-group protagonist threatened immediate personal interests, and those of fellow in-group members, and we observed these varying degrees of threat to have meaningful impact on human cooperation and competition. The decision making was one-shot and private, rendering moot the motivation to acquire quality signals from which one benefits in the long-run. As such, the behavioral game developed here provides a useful step towards disentangling self-interested versus group-serving motives underlying human cooperation, ultimately providing an empirical answer to the penultimate question whether humans are, indeed, a cooperative species [53], [54].

Participants engaged in decision making without feedback. In as much as cooperation tends to elicit cooperation, non-cooperation often begets non-cooperative responses [11], [55]. From this it follows that the defense-motivated non-cooperation induced by oxytocin may provoke non-cooperative responses in the out-group protagonist, potentiating a vicious spiral of increasingly competitive exchange. This would be particularly likely when non-cooperation is excessive, as we observed here when individuals were given oxytocin and fellow in-group members were vulnerable. Accordingly, oxytocin appears to direct individuals towards their in-group, motivating not only trust and generosity towards the in-group, but also strong motivation to protect and defend the in-group against outside threat. Inadvertently, such oxytocin-modulated tend-and-defend tendencies may trigger non-cooperation in outsiders, and create the very threat that individuals under oxytocin tried to ward off.

## Materials and Methods

### Participant Recruitment and Test Medication

Males (*N* = 105; Mean age = 21.13 years, *SD* = 1.29) were recruited via an on-line system and offered €10 (approx. USD 13) for participating in a study on medication and decision-making. Exclusion criteria were significant medical or psychiatric illness, prescription-based medication, smoking more than five cigarettes per day, and drug or alcohol abuse. Data of three participants were discarded because they erred on all six questions verifying their understanding of the experimental game. The experiment was approved by the Psychology Ethics Committee of the University of Amsterdam, and adhered to the guidelines set forth in the Declaration of Helsinki. Participants provided informed consent prior to the experiment.

Participants were instructed to refrain from smoking or drinking (except water) for 2 hours before the experiment and randomly assigned to the oxytocin or placebo group (double-blind, placebo-controlled study design). Participants self-administered a single intranasal dose of 24 IU oxytocin (Syntocinon-Spray, Novartis; 3 puffs per nostril) or placebo. The placebo contained all the active ingredients except for the neuropeptide, was prepared according to Good Manufacturing Practice (GMP) and Good Clinical Practice (GCP), and delivered in the same bottles as Syntocinon.

### Experimental Procedures

Participants came to the laboratory and learned that pay was equivalent to their show-up fee and their earnings during the decision task (range €1—€8). Data were collected in Spring 2011 (*N* = 75), and in Spring 2012 (*N* = 30). Analyses revealed no interaction effects involving data collection wave on any of the dependent variables and this factor is further ignored. Participants were seated in individual cubicles preventing them from seeing others and communicating, signed an informed consent, and self-administered the medication under experimenter supervision. The experimenter left and participants completed a series of unrelated questionnaires and tests that were presented on their computer screen, using the keyboard to answer questions.

Because effects of oxytocin plateau after approximately 35 min [26] and in keeping with earlier studies [17], [27], the computer switched to the experimental instructions after 30 minutes. Participants read that they would engage in a decision making task involving the participant's own group (denoted as “Triangle”), and another group (denoted as “Circle;” labeling was counterbalanced but had no effects and is further ignored). Groups were composed on the basis of the order in which participants had signed up for the experiments, so that most but not necessarily all involved individuals were currently present in the laboratory. They were also told that each group contained three members, and that they would not know who was in their group or who was in the other group.

Participants then read that their income to “Triangle” (their in-group) and to “Circle” (the out-group) depended on the participant's own decisions, and those of a representative of Circle's whom they would be paired with through the computer network. A table showed the payoffs to Triangle (in-group) and Circle (out-group) as a function of the four possible combinations of choices (1 or 2 by in-group; 1 or 2 by out-group, with 1 = Cooperation, and 2 = Non-cooperation). We altered the cardinal payoffs to create variation in Personal and In-Group Vulnerability while at the same time maintaining the ordinal structure of the game as well as Greed and Cooperator's Gain (see main text). This resulted in four different games (see Fig. 1B—1E, main text), and participants were randomly assigned to one of these.

After being fully trained on the decision game, participants answered quiz questions (analyses showed that all participants except three—whose data were discarded—understood the game and the implications of their choices, and that understanding did not vary across conditions), were assured that decisions would remain confidential, and asked five times to make a choice between 1 (cooperation) and 2 (non-cooperation) (no feedback was given). To motivate serious decision making, participants were reminded prior to each choice that this choice might be randomly selected for pay-out to all members of Triangle and Circle. As participants interacted through the computer network they could not see each other. Following decision making, we assessed in-group tending (“during decision making I tried to serve the interests of my group” and “during decision making I tried to help my team”) (always 1 = not at all, to 7 = very much) and in-group protection (“when making decisions, I tried to protect my own group,” and “during decision making, I tried to prevent losses to my own group). Due to a programming error, data on in-group protection were recorded only for the Spring 2011 wave. The main experimental task (instructions, decision making, and post-task questionnaire) took 14—17 min. Upon finishing the questionnaire, participants were thanked and dismissed. They returned four to six weeks later to pick up a sealed envelope containing their credits, additional earnings, and a full debriefing. We used a time-lag between experimental session and debriefing to prevent that any insight on methods and materials became known before the entire experiment was completed. All participants agreed to this procedure.
